# Deterioration of Electrical Load Forecasting Models in a Smart Grid Environment

**DOI:** 10.3390/s22124363

**Published:** 2022-06-09

**Authors:** Abdul Azeem, Idris Ismail, Syed Muslim Jameel, Fakhizan Romlie, Kamaluddeen Usman Danyaro, Saurabh Shukla

**Affiliations:** 1 Electrical and Electronics Engineering Department, Universiti Teknologi PETRONAS, Seri Iskandar 32610, Malaysia; fakhizan.romlie@utp.edu.my; 2 Postdoc Scientist at Structure Lab, School of Engineering, National University of Ireland Galway (NUIG), Galway H91 TK33, Ireland; muslimjameel.syed@nuigalway.ie; 3 Computer Science Department, Universiti Teknologi PETRONAS, Seri Iskandar 32610, Malaysia; kamaluddeen.usman@utp.edu.my; 4 Data Science Institute (DSI), National University of Ireland Galway (NUIG), Galway H91 TK33, Ireland; saurabh.shukla@nuigalway.ie

**Keywords:** energy management, adaptive models, generation modalities, load forecasting, machine learning, model deterioration, power stability, Smart Grid

## Abstract

Smart Grid (S.G.) is a digitally enabled power grid with an automatic capability to control electricity and information between utility and consumer. S.G. data streams are heterogenous and possess a dynamic environment, whereas the existing machine learning methods are static and stand obsolete in such environments. Since these models cannot handle variations posed by S.G. and utilities with different generation modalities (D.G.M.), a model with adaptive features must comply with the requirements and fulfill the demand for new data, features, and modality. In this study, we considered two open sources and one real-world dataset and observed the behavior of ARIMA, ANN, and LSTM concerning changes in input parameters. It was found that no model observed the change in input parameters until it was manually introduced. It was observed that considered models experienced performance degradation and deterioration from 5 to 15% in terms of accuracy relating to parameter change. Therefore, to improve the model accuracy and adapt the parametric variations, which are dynamic in nature and evident in S.G. and D.G.M. environments. The study has proposed a novel adaptive framework to overcome the existing limitations in electrical load forecasting models.

## 1. Introduction

The current developments and modifications in electrical networks and infrastructures, including distributed energy resources (D.E.R.s), have supported the energy demand. These advancements have helped clean power production through renewables integration and have also increased electrical power systems’ complexity [1]. It has triggered several complications in electrical forecasting due to the diversity involved in power production and each power source’s distinct behavior concerning different variables over time. This diverse phenomenon of energy production has challenged the electrical load forecasting (E.L.F.) models to perform power predictions accurately. However, due to existing complications, it always results in a certain percentage of error with the forecast.

Since E.L.F. is responsible for meeting the demand-supply gap, their accuracy is of utmost importance and paid immense attention [2]. Many factors cause this occurrence of results with errors inaccuracy. Some are due to incomplete data, noise, power surges, inappropriate consideration of forecast factors, applications, appropriate model selection, and parametric tuning [3].

In E.L.F., short-term load forecasting (STLF) is a driving factor of day-to-day operations. It impacts operations, financial costs, and savings, which are considered essential. Several STLF models have been proposed using statistical, regression, and artificial intelligence (A.I.) [4,5]. Based on the performance of these models, several hybrid models were also introduced to improve the forecasting accuracy; these models included several techniques with clustering and optimization algorithms. However, the models pose limitations to the changing environment due to their static behavior.

In a Smart Grid (S.G.) environment, the electrical consumption patterns keep changing. These changes are observed due to different factors, but not limited to weather, processes, activities, and events. While these factors are not cyclic, the consumption pattern of electrical load differs from one hour to another throughout time. Since the data streams in the S.G. environment are continuous, pattern changes are observed on a large scale. Numerous E.L.F. models have been proposed, but when new and unknown parameters influence the electrical consumption pattern, the conventional machine learning (ML) models cannot observe the changes and stand obsolete to handle such issues. These models can be differentiated as below:

Traditional/Shallow learning models: The shallow learning models (S.L.M.) can be described as the models designed for dedicated applications, industries, utilities, sectors, and regions. These models have a pre-defined set of features and functions that cannot update, change, or modify themselves over the requirement. Any change requirement needs to be set manually and offline for each change operation. Therefore, these models stand obsolete in continuous data streams in the S.G. environment. Thus, the deep learning (DL) models have replaced them. Still, it is challenging for researchers to discover new ways to improve precision and accuracy during the forecast and make models adaptive. Despite many DL methods proposed by different researchers, the accuracy and adaptability factors are still unsatisfactory.

**Figure 1:**
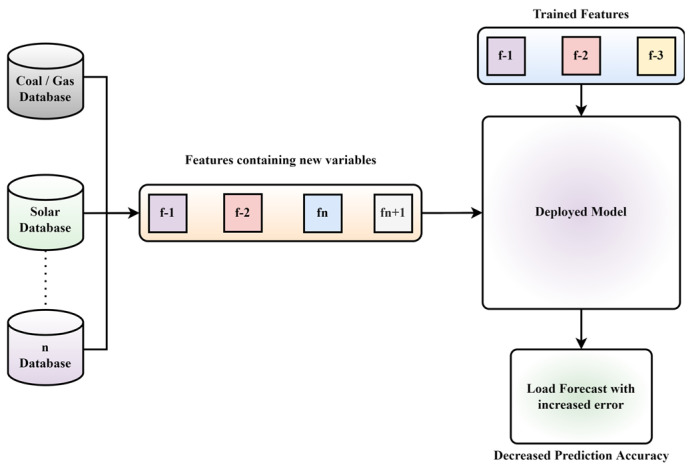
An illustrative example of deterioration in prediction accuracy of traditional models on introducing new parameters.

Back draw of Traditional Models: The recent machine learning models for electrical load forecasting comprise a set of trained features, and the same elements are kept constant while testing. Therefore, the models keep specific acceptable results until the input feature or application changes. However, when we observe a single or multiple changes in the input features in an online system, the model starts to deteriorate and produce poor forecasts. Such forecasts impact energy management and thus cause a loss in terms of millions of dollars in operations and maintenance. Figure 1 describes a scenario where a deployed model is trained on selected features (f-1, f-2, and f-3). However, introducing new features (fn and fn+1) deteriorates the model performance.

The change in feature could occur due to a change in database, application, sector, or modality since the features selected in recent ML models are not standardized for any particularity. Therefore, their importance could change with respect to time. Moreover, the electrical pattern observes continuous change depending on different factors. Therefore, considering those factors are of utmost importance, and their integration as essential features needs to be considered while designing the model.

It is observed that different parameters have a different impact on load forecasting. This impact needs to be calculated before involving any parameter since it can either increase/decrease the accuracy or cause complexity in the model performance.

Adaptable Machine Learning Model: Adaptability is a feature that transforms any machine learning model to adjust itself and perform self-regulations following the environment. This is a self-regulatory attribute responsible for dynamically adjusting the model concerning changes in data trends. These ML models can retain their precision and accuracy even after parameter, application, utility, region, and sector variations. The adaptability approach is categorized into the semi- and fully-adaptive approaches, with few dynamic changes by fundamental structure improvement and autonomous module incorporation for self-tuning as per required features.

The existing ML models have the nature of modeling static data. Therefore, these models cannot capture the dynamic changes observed in input parameters for load forecasting in the S.G. environment and cannot update themselves automatically (neither the features nor the model). This directs them to generate poor forecasts, have model limitations, and have an inconsiderable error percentage in the results, causing a loss in terms of cost for operations and generations [6]. 

The electrical consumption patterns in S.G. and D.G.M. are acyclic, for which the model needs to be updated automatically with new features and patterns. These changes in input parameters need to be automatically updated in the model because the parameter tuning could not be performed physically in a real-time environment. Therefore, along with the discussion of the deterioration of E.L.F. models, we have also proposed an adaptive model framework for our future works, which can update itself for variations in parameter, application, utility, region, and sector utilizing self-adaption strategies. 

The proposed framework can entertain the S.G. and D.G.M. environment changes, perform real-time correlation with different parameters, and utilize the input parameters that have a more influential impact on the load while discarding the irrelevant features. The difference between the previous and our proposed model lies in model adaptation, modality recognition, parameter defining concerning modality, and continuous up-gradation of the model with its relevant features in a real-time environment.

In this paper, we have examined the performance deterioration of existing load forecasting models concerning S.G. and D.G.M. environments, considering simple parametric variations to support the theory and proof the same with the help of experimentation. The study confirmed the performance deterioration of forecasting models on three different datasets. The evaluation is based on forecasting results produced by the models conducted before and after the change in input parameters. Based on the experimental results and our observed limitations of existing load forecasting models. The study has proposed a novel adaptive framework to overcome the existing challenges experienced in S.G. and D.G.M. environments. 

The main contributions of this study can be summarized as follows:

1. To highlight the performance deterioration in E.L.F. models due to changes in parameters and modality.
2. To highlight the importance of STLF in the S.G. and D.G.M. environment.
3. To evaluate existing load forecasting models.
4. To propose a novel adaptive framework to improve the model deterioration in E.L.F.
5. To emphasize the importance of adaptive models in real-time E.L.F.

The study discusses the limitations that cause the model deterioration, and a novel adaptive framework is proposed to improve the model deterioration since conventional models are obsolete in such a dynamic environment. The proposed model is believed to contribute to the D.G.M. and S.G. environment, where operation and power management are susceptible tasks. The proposed framework is crucial to assist utilities, independent power producers (I.P.P.), industries, off-grid consumers, and Smart Grid management in forecasting energy demand. It is also believed to help manage it through efficient adaptability features to integrate D.G.M. and manage power under respective elements affecting the power generation. Thus, intelligent energy management will be enabled through the proposed novel framework to direct an intelligent implementation of D.E.R.s. It will help with electricity planning, generation, transmission, distribution, scheduling, operations, and efficient integration of multiple power sources for clean optimum power generation and consumption. 

The rest of the paper is distributed as Section 2 reviews the literature of related works and previous propositions according to the proposed framework and conventional modeling and their limitations, including traditional and adaptive load forecasting models. Section 3 provides the methodology of model evaluations and their deterioration and dataset descriptions. Section 4 provides the results and discussion of model deterioration. Finally, Section 5 concludes the study and future research directions and work propositions.

## 2. Literature Review

Based on the intensive literature review, we conclude that the traditional methods deployed for load forecasting and associated factors such as generation, transmission, distribution, and operations and planning would require a more robust forecasting solution to be deployed for S.G. and D.G.M. In the era of continuous technological evolution, one hundred and ninety-three countries at the United Nations have agreed upon the agenda of the sustainable development goals (S.D.G.s). Out of these goals, the smart city is of crucial importance since it is estimated that today 54% of people live in cities worldwide, but by 2050 this proportion is estimated to reach 66%. The cities are already facing the issues such as power management, unsustainability, and some other fragile problems [7]. In such scenarios, the smart city, with its advanced technologies (comprising I.C.T. and innovative advances) addresses these issues (providing efficient power management, improving quality of life, promoting economic growth, and developing a sustainable and safe environment). However, the foundation of a smart city lies with the implementation of S.G.s that can efficiently support technological advances.

**Figure 2:**
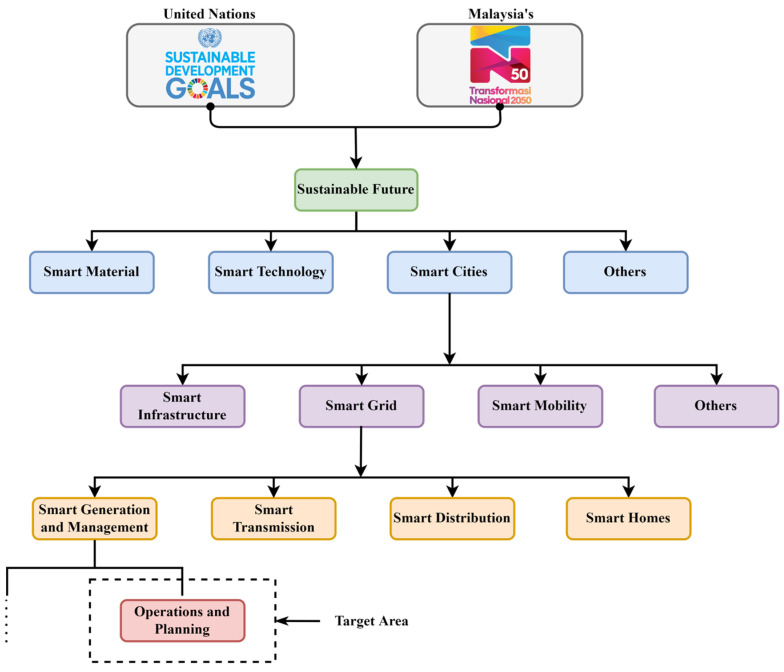
Hierarchical structure highlighting the importance of target area in smart cities and Smart Grid for research impact on sustainable future.

The S.G. is comprised of D.G.M., which could include D.E.R.s. This further integration of different power resources supports system availability and increases robustness, but the system complexity also increases. Though the S.G. is an unprecedented opportunity to adapt IR 4.0 and update the energy industries into the era of reliability, availability, and efficiency that contributes to intelligent power systems [8], E.L.F. accuracy is one of the critical issues S.G. to forecast the future demand accurately. Figure 2 presents a hierarchical structure highlighting the importance of the target area in smart cities and Smart Grids for research impact on a sustainable future leading towards the efficient generation and management of electrical power.

E.L.F. helps control and manage energy generation and reduces the behavior of energy fluctuation [9]. Since load forecasting deals with minimizing the utility risk through forecasting the future demand, it is one of the most crucial areas in implementing a Smart Grid and creating the foundation of a smart city. Still, due to the dynamic behavior of load in the S.G. environment, the current models either stand obsolete or rapidly deteriorate their performance.

Though the transition period from conventional grids to Smart Grids is critical, the benefits are rewarding. Some of the benefits include:

- Efficient generation, transmission, and distribution of electricity,
- Power acquired from different generation modalities,
- Fast network restoration (in case of any disturbance),
- Operations and management cost reduction for utilities,
- Reliable integration of large-scale renewables.

Since S.G. is dependent on accurate and efficient load forecasting, E.L.F. is treated as a critical operational task. Thus, it has been distributed into four categories concerning the forecasting time-domain long-term load forecasting (LTLF), medium-term load forecasting (MTLF), short-term load forecasting (STLF), and very short-term load forecasting (VSTLF) [10]. Several forecasting methods have been employed for these time domains, including the knowledge base expert system and some statistical techniques, artificial intelligence (A.I.) techniques, machine learning (ML) models, and hybrid techniques.

**Figure 3:**
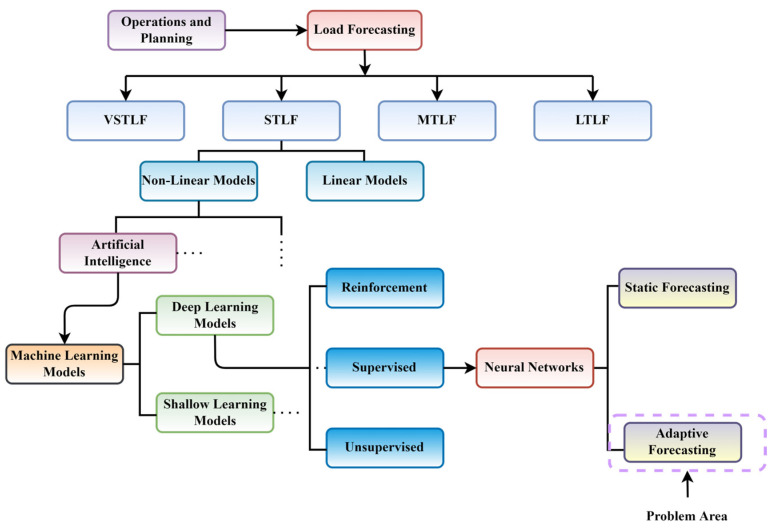
Hierarchical structure highlighting the existing problem areas of S.G. and D.G.M.

In S.G. and D.G.M. environments, the STLF (time zone ranging from a few minutes, hours, or days) is a significant factor in day-to-day operations and planning of a power utility and critical component of an energy management system. The STLF approach is efficient in reducing financial costs and operational risks, and it impacts directly on savings. Therefore, it is given much prominence and treated as a critical problem in the competitive energy market [11,12]. Figure 3 shows the hierarchical structure leading from the Smart Grid to this study’s load forecasting problem area.

Several STLF techniques have been proposed, some of which have been classified as statistical methods, including linear regression, auto-regressive moving average (ARIMA), and exponential smoothing (E.S.) [13]. Later in STLF, scientists introduced specific new methods, including artificial neural network (ANN), fuzzy logic (F.L.), support vector machine (SVM), recurrent neural network, and long short-term memory (LSTM) [12,13].

Numerous statistical, machine learning, and deep learning models have been proposed in the literature for different forecasting applications [14]. Still, they have various limitations in their design, architecture, application, region, sector, etc. Some current methods proposed for different forecasting applications claim better accuracy but stand obsolete in dynamic environments [15]. The traditional models are discussed below:

### 2.1. Traditional Models

Some of the traditional load forecasting models include [16] where Tina, G.M. et al. presented a state of the art review on ML-based methods for P.V.s, anomalies, fault, and optimization detection and analysis. The review discussed several past studies of machine learning algorithms used explicitly in P.V. generation, forecasting, and fault detection and their pros and cons. Khodayar M. et al. [17] explored different neural networks (LSTM, CNN, and G.R.U.) to forecast P.V. energy with the help of optimization dictionaries to improve the forecasting results.

Rai, S. and De, M. [18] presented their analysis of different ML-based forecast models relating to S.G. The work is dedicated to the N.I.T. Patna campus in India for STLF and MTLF. The data was attained from the smart meters, where the claimed dataset is said to be of multiple load types. However, considering the institutional operations, the dataset is assumed to be cyclic. However, multi-node forecasting is an efficient process considered for forecasting in the study. Deiss, M. B. et al. [19] compare the difference in electrical load between office buildings and residentials during the COVID-19 pandemic. The study provided the power consumption comparison while working at the office and work-from-home scenarios, presenting the hypothesis of increment and decrement of energy consumption in residential and commercial buildings in case of a pandemic.

Wu, T. and Wang, J. [20] discussed the artificial intelligence (A.I.) applications for the operation and control of micro-grid (M.G.), emphasizing the deployment of deep learning (DL) and deep reinforcement learning (DRL) for power applications, including distributed energy resources in utilities. Wang, N. et al. [21] have explored the problems of prosumers for energy trade through distributed ML applications focusing on the issues of data privacy, limited communication, and computation. It focuses on developing a framework that provides intelligent energy management for residential prosumers and allows them to trade energy in the local vicinity. The study has proposed an ML-based framework for optimizing load, accuracy of price forecast, and improved efficiency for trading energy for a direct current (D.C.) grid.

Hafeez, G. et al. [22] proposed a hybrid model for STLF composed of modified-mutual-information (MMI) for tailoring the input features, factored-conditional-restricted-Boltzmann-machine (FCRBM) for predicting day and week demand, and genetic algorithm along with wind-driven optimization (GWDO) for fast convergence. The proposed model was tested on power grids of the U.S.A. Motepe S. et al. [23] proposed an A.I. and DL-based method for load forecasting to prepare maintenance and operations at the distribution network. The study has also conducted a comparative survey of the current state-of-the-art methods where LSTM was observed to provide better forecasting accuracy than ANFIS and OP-ELM. However, the study is only related to South Africa, and the data’s spikes, dips, and sags were removed. Such removals will expose the model to poor accuracy in real-time environments. 

Zheng J. et al. [24] proposed an analysis for considering multi-variable data based on copula correlation to determine the optimal parameters and used the improved LSTM methodology to attain better load forecasting accuracy. The factors affecting the L.F were trained back and forth to train the model, resulting in comprehensively better accuracy. Farsi B. et al. [25] presented an approach of ML algorithm by combining LSTM and CNN in parallel to improve the forecasting results. The study considered the datasets from Malaysia (hourly load) and Germany (daily load) to perform STLF and claims to improve the forecasting accuracy considerably. 

Bouktif S. et al. [26] modeled a framework using G.A. for parametric optimization and LSTM for E.L.F. based on STLF and MTLF. The study involved the R.T.E. dataset from France metropolitan’s electricity, and the modular development was univariate, making the proposed model obsolete in a dynamic environment. S.G. Ghadimi, N. et al. [27] presented a two-stage forecasting engine comprising R.N.N. and E.N.N. and a feature selection technique making the model more competitive to consider only appropriate variables and filter the rest.

Azeem A. et al. [6] presented a comprehensive review of different load forecasting models concerning different sectors. The study has also exploited the related models under residential, commercial, industrial, grid-connected, and off-grid sectors. It also covers the importance of different generation modalities in the modern environment of Smart Grids. It discusses the limitations of existing load forecasting models concerning the implementation of S.G. and the behavior of electrical load concerning different factors affecting load forecasting. Potocnik, P. et al. [14] compared different ML forecasting models to determine the future natural gas demand for urban areas. The forecast variables consisted of temperature, time, holidays, and events. The forecasting criteria cater to various horizons; however, it concluded linear regression and recurrent neural networks fit different criteria. 

Jahan, I. S. et al. [28] reviewed different load forecasting models, sectioning their findings into groups of ANN, SVM, D.T., L.R., and F.S. It also discusses the critical factors that affect load forecasting over the period. Yang, Y. and Wu, L. [29] discuss the power system problems and their respective existing solutions, referring to unit commitment (U.C.). The study examined several ML approaches for solving the issues in nonlinear A.C. power flow. Guo W. et al. [30] discussed the three most prominent ML algorithms of load forecasting (SVM, R.F., and LSTM) and presented a model by fusion of these methods for improved STLF. Moreover, a comparative study to support the performance of the proposed model is also given.

### 2.2. Adaptive Models

When the load forecasting environment kept improving, new D.E.R.s were introduced to the power generation system. It enhanced the generation capability but also increased the system complexity. Several adaptive models were proposed to accommodate those complexities, including [15], where Ding S. et al. proposed a model for power generation of photovoltaics utilizing the nonlinearity, periodicity, and fluctuation of parameters over time and optimizing the solution. The model supposedly forecasts the P.V. generation, including more complex long-term forecasting parameters. The study proposed a novel adaptive model with time-varied parameters incorporated with the help of G.A. The study claims to adapt the parameters but has not covered the aspects of sparse, noise, loss, and data fluctuations, including new parameter introduction. However, the considered parameters and their variations are a subset of total parameters compared to S.G. and D.G.M. environments since P.V. is a subset of D.G.M.

Mohammed, N.A. and Al-Bazi A. [31] proposed an improved ANN along with an adaptive backpropagation algorithm (ABPA) to cover the limitations of ANN and provide enhanced forecasting accuracy. The datasets comprised nine years of data attained from the ministry of electricity in Iraq. The study considered different vital energy factors and integrated them into the training to improve the forecast.

Wu, S. F., and Lee S. J. [32] presented a strategy for the local modeling strategy of ML algorithms consisting of N.N.s, adaptive neuro-fuzzy inference system (ANFIS), and least square SVM (LSSVM) to improve the forecasting performance of in predictions relating to time series. The study has posed to achieve a target that was set prior. However, in cases of the acyclic nature of data, Smart Grid, and multi-modality generations, such initial marks are irrelevant since they pose higher degree errors. 

Laib O. et al. [33] presented a two-stage predictive approach that consists of an adaptive hybrid forecasting model to predict the natural gas consumption using LSTM for prediction and M.L.P. for profiling the next day’s gas consumption in Algeria.

S-Medina, J. J. et al. [34] proposed an adaptive model based on incremental linear regression where the model on arrival continuously learns the new streams, and a new window is generated to learn based on past windows. Though the model consistency was a strength, the linearity proposition is unsuitable for highly nonlinear environments such as S.G. and D.G.M.

Guo T. et al. [35] discussed the importance of streaming data in several real-time applications. They proposed a method based on the adaptive R.N.N.s approach utilizing the gradient method for learning. Since in STLF, we need good data forecast, but the model only supports a step-ahead prediction.

Parameter optimization and selection of suitable models are critical when dealing with E.L.F. Some other studies that proposed parameter optimization and dimension reduction to improve the implication of critical parameters were also presented [36,37]. Still, it is deduced that most of the studies have praised the importance of using R.N.N.s and their significance in dealing with time series problems since it outperforms others [38,39].

### 2.3. Adaptive Models with Concept Drift

In the current challenging environment, the evolution of data occurs over time. The patterns and associated correlations evolve, and this phenomenon in ML is termed concept drift. A similar phenomenon occurs in dynamic electrical environments, which involves S.G. and D.G.M. Some other studies considering the concept drifts are:

Jagait, R. K., et al. [40] presented an ensemble approach for online learning using R.N.N.s and ARIMA. The study proposed an adaptive model based on concept drift using R.N.N.s and considered ARIMA for creating a rolling window operation to create an online scenario for the ensemble model. Though the model has achieved considerable improvements, the model cannot be considered acceptable to be implemented in dynamic environments, especially Smart Grids and with multi-generation modality, since the rolling window operations have known values without any noise or loss. Still, real environment scenarios differ. The same goes with the features improvement and consideration in D.G.M. environments where the feature continuously changes with modality.

Madireddy S. et al. [41] proposed a model under concept drift for scheduling jobs in production. Kracnnichfeldt, L. V. et al. [42] purported a model based on the approach of Passive Aggressive Regression (P.A.R.). The utilized technique included individual and ensemble forecasts learning to support model adaptivity. However, nonlinearity, complexity, and exclusion of feature adaptivity make the model vulnerable to S.G. and D.G.M. 

Fekri, M.N. et al. [43] presented an approach considering online Adaptive-RNN with batch normalization technique with R.N.N. Lalis J.T. and Maravillas E. [44] presented a dynamic model for load forecasting using an adaptive system considering multiplayer and perceptron involving minimum complexity. Ammar N. et al. [45] propose a model with adaptivity utilizing neuro-fuzzy-inference (ANFIS) to analyze the impact of different weather parameters on current and previous electric loads.

Nalacaci G. et al. [46] utilized a multivariate adaptive regression spline (MARS) model and compared it with ANN and L.R. to produce LTLF and deduced that result of MARS was improved in comparison to others. Xiaolan, L. and Zhou, J. [47] combined the strategies of parametrization, fractal interpolation function, iterative learning, and chimp optimization algorithm (CLFIF-IL-ChOA). They later compared it with statistical and other machine learning algorithms, deducing that the model performs better in an adaptive environment. Zhang, Y. et al. [48] proposed a method for E.L.F. prediction interval based on reinforcement learning with adaptivity to address probability–proportion selection and quantile-forecasting.

Among the literature survey, Jameel S. et al. [49,50,51] proposed multiple adaptive frameworks in their studies. They presented an adaptive framework for different ML and DL applications that included complex and multispectral image analysis, image classification following the digital transformation of IoT and IR 4.0, and disease identification in skins to detect it at an early stage.

From the literature, we can safely state that the load forecasting models face accuracy challenges due to parametric variations causing irregular consumption patterns, and non-or-partial adaptability in the models is a critical problem [52]. Some recently proposed adaptive models’ strengths have also been discussed in Table 1. Since the current models perform on few parameters and if new changes are introduced, the model performance degrades gradually. To conclude, we will analyze various load forecasting approaches and their learning strategies in a later section and adopt the best method to improve the structure dynamically. Hence, a significant contribution of this study is to introduce an adaptable model framework for continuous data stream in a Smart Grid environment to improve the accuracy of load forecasting models.

### 2.4. Brief Review of Some Load Forecasting Methods

**Figure 4:**
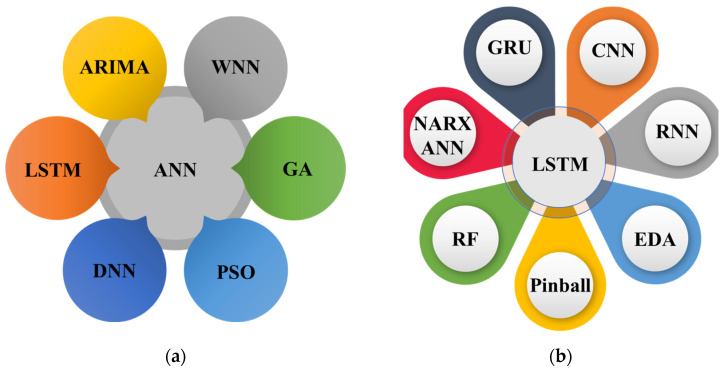
Different ensembles of E.L.F. models used in literature: (**a**) ANN; (**b**) LSTM.

The literature reveals many approaches used for load forecasting, whether as a single method, hybrid method, mixture of different techniques, or clustering and optimization methods for improved electrical load forecasting results. After an in-depth literature review, we found some most prominent load forecasting approaches used in literature either in singular form or with different techniques. It is also highlighted in Figure 4a, where ANN was combined with ARIMA, wavelet neural network (W.N.N.), genetic algorithm (G.A.), particle swarm optimization (PSO), deep neural network (D.N.N.), and LSTM. Similarly, Figure 4b presents different combination used with LSTM that includes gated recurrent unit (G.R.U.), CNN, R.N.N., estimation distribution algorithm (E.D.A.), random forest (R.F.), and nonlinear auto-regressive exogenous (NARX) model. This section briefly reviews the most prominent approaches used in numerous studies relating to electrical load forecasting.

#### 2.4.1. Artificial Neural Networks

**Figure 5:**
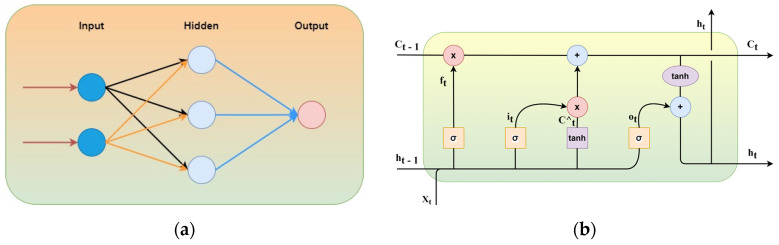
The basic architecture of (**a**) Normal ANN with two inputs and a single hidden layer; (**b**) LSTM.

ANN has apprehended numerous applications due to their high skill in learning. These methods are popular for forecasting for their reliability and accuracy. An estimation of function could be performed using ANN. It consists of layers termed input, hidden, and output, shown in Figure 5a as a primary architecture model. The layers consist of nodes equal to variables existing in each layer, respectively [53]. Input layer and parameter (N.F.) are equal, represented in the size of the column vector of [NFx1], *x_t_*, where “*t*” is the time constant. The number of hidden layers depends on the methodology.

For a single hidden layer consisting of *N_H_* weighting a matrix of *W_H_* with a bias *b_H_* vector, using activation $\rho_{H}\;$ function, the hidden layer output could be expressed as:

(1) $$y_{t}^{H}=\rho_{H}\left(W_{H}x_{t}\right)+b_{H}$$

Treating its input for the final output we receive:

(2) $$y_{t}^{o}=\rho_{o}\left(W_{o}y_{t}^{H}\right)+b_{o}$$

$y_{t}^{o}\;$ output size: $\rho_{o}$ is the function of activation and $\;W_{o}$ is weight matrix. *T_o_* minimize the error criterion, the ANN throughout training changes biases and weights, which can be expressed as below function:

(3) $$min_{W,b}\frac{1}{2}\overset{T}{\underset{t=1}{\sum }}{\left(y_{t}-y_{t}^{o}\right)}^{2}$$

#### 2.4.2. Long Short-Term Memory

One of the deep recurrent neural network family members is LSTM, used in various models such as time series, recognition of speech, and language. LSTM models have outperformed other methods for learning from earlier stages which is vital for the future forecast. In contrast to a feed-forward neural network, LSTM consists of cycles of network activation, which feeds from the recent step as a network input, influencing current time predictions [54]. Consequently, the model develops its memory from previous events and is in its hidden state variables encoded. As shown in Figure 5b, the system takes *x_t_* as input at the current time, *h_t_*_−1_ as output from prior LSTM, *C_t−_*_1_ as previous unit memory, *h_t_* as current system output, *c_t_* as current unit memory, it as model input, ot as model output, and *f_t_* as forget gate.

The input gate decides to add info from the present input state of the cell. The forget gate’s decision includes removing the report from *h_t−_*_1_, only keeping the most relevant information, and the output gate takes a decision regarding out info from the current cell state. The equations controlling the cells of LSTM are as follows:

(4) $$i_{t}=\left(W_{i}.\left[h_{t-1},\;x_{t}\right]+b_{i}\right)$$

(5) $$f_{t}=\sigma \left(W_{f}.\left[h_{t-1},\;x_{t}\right]+b_{f}\right)$$

(6) $$o_{t}=\sigma \left(W_{o}.\left[h_{t-1},\;x_{t}\right]+b_{o}\right)$$

(7) $$\tilde{C}=tanh\left(W_{c}.\left[h_{t-1},\;x_{t}\right]+b_{c}\right)$$

(8) $$C_{t}=\left(F_{t}*C_{t-1}+i_{t*}{\overset{ˇ}{C}}_{t}\right)$$

(9) $$h_{t}=o_{t}*\tanh\left(C_{t}\right)$$

*C_t_* is the cell state, *h_t_* is the hidden state, and *σ* is the sigmoid function. Thus, the system performs computation that decides the output and is dependent on input, past values, and past analyses, enabling the model to grasp different time-scaled information for current computation purposes.

#### 2.4.3. Auto-Regressive Integrated Moving Average (ARIMA) Model

When the data perceived in a process is not stationary, it must first be transformed into a static form. The variance in the ARMA and ARIMA model transforms time series into static by d-order difference before fitting the series (d represents order difference). ARIMA also has issues such as over or underfitting data due to *p* and *q* values. A criterion, namely B.I.C., is used out of a set of models to select the optimal model used to reduce uncertainty and improve prediction accuracy. The lowest B.I.C. score is chosen as the best fit model [55].

(10) $$BIC=k\times \ln\left(m\right)-2\ln\hat{\left(L\right)}$$

where ‘*k*’ represents the number of model parameters, ‘*m*’ sample size, and ‘*L*’maximizes the value of the function. Though the ARIMA model was modified with the help of B.I.C., it is not yet adequate to accommodate the electrical load without its combination with some other method that can change the absence of data compensation [56].

It is evident from the literature and our experimental results that most load forecasting models are static and cannot adapt to the changes. The changes could be for parameters, application, sector, region, modality, etc. Due to this deficiency, they do not improve their model accuracy and are dedicated to only designing static applications. Therefore, such models need manual modifications to update themselves whenever a new feature is introduced. Besides, every application has a dedicated model, which stands obsolete in a different environment. Moreover, the existing literature lacks in addressing a model which encompasses different generation modalities, fully adaptive forecasting, and fully adaptive real-time forecasting. A model that can accommodate the changes in input features, applications, modality, and regions can be sufficient for the S.G.M. and D.G.M. environment and possess the dynamic ability to enhance the model’s ability to adapt to multi-variable online and real-time environments. 

Therefore, such models are required can adapt themselves automatically without interruption and manual modifications. Considering such capabilities, we have proposed a novel framework to address the issues and challenges experienced by existing load forecasting models. The proposed approach is designed to obtain data from multiple sources. The data is then deployed to the classifier and outlier sub-module in its feature identification module to fulfill the initial D.G.M. requirements. Later, to record the old and new changes, a repository is formed which captures dynamic features and constantly compares itself with identification and classification to fulfill S.G. and D.G.M. requirements of continuous change interpretation.

Furthermore, the online training module consistently receives the input from the output. The LSTM ensemble is designed in a multi-layer parallel working mode that evaluates the forecast and generates optimum results. Thus, all incoming data is assessed for new data, features, and other changes to be adapted if observed, making it a novel framework. The description of the proposed framework, its modules, and their working is detailed in the methodology section.

The existing models cannot adapt to the dynamic changes observed in S.G. and D.G.M. environments. Such as input feature changes in a real-time deployed environment and observed differences following region, modality, sector, and application. Thus, these models stand obsolete in such vibrant scenarios. Therefore, due to its ensembled module capabilities and novelty of continuous adaptivity in features and observed changes in its environment, the proposed model stands unique in comparison to existing models. It is deduced that the traditional models stand obsolete, and the proposed adaptive models face various challenges to contend in such a dynamic environment. They pose challenges for accuracy and precision, effective energy management, planning and operations, and future load prediction. Therefore, it generated the need to investigate the recent models concerning the challenges encountered in the S.G. and D.G.M. environment, observe the model’s behavior, and produce forecasts with different variable shifts and parameter changes.

To overcome the described adaptability challenges, this study is crucial to explore the E.L.F. models and observe their performance in a distinct environment where the data has multiple changes in terms of modality and parameter. Moreover, it discusses the existing challenges and the proposition of a new framework to overcome the current issues in E.L.F. models following S.G. and D.G.M.

## 3. Methodology

This section explores the existing load forecasting models regarding S.G. and D.G.M. and elaborates on the proposed tentative adaptive framework. The intuition behind this framework is to adapt the modality and variations in parameter and region features. It is evident from the literature that to achieve accuracy in E.L.F. models, the data under observation shall be free of noise, loss, and error to attain acceptable results. In this study, we have considered two open-source datasets provided with the name of American electric power (A.E.P.) ranging from 2004 to 2018 and New York City (N.Y.C.) ranging from 2012 to 2017. The third dataset belongs to the real environment of Universiti Teknologi PETRONAS (U.T.P.), ranging from Nov to Dec 2019The dataset comprises “hourly load data” in megawatts (M.W.s) but have different parameters.

Due to the involvement of unidentical parameters, we have observed the change of performance in model accuracy with the evolution of parameters. Thus, the difference in parameters has caused the models’ performance deterioration. The study uses ARIMA, ANN, and LSTM models on each dataset. Therefore, the study is subdivided into three case studies for better understanding and description.

### 3.1. Dataset Description

The utilized database comprises three datasets; two of them are available online at Kaggle with descriptions provided below. Since the load data is time series, the analysis of such vital attributes is required. To observe those attributes, we have decomposed our datasets into ‘trend’ showing the stability and instability of data, ‘seasonality’ expressing the fluctuations occurring at a certain periodicity, and ‘residual/noise,’ which depicts the remains. Further details are discussed below:

#### 3.1.1. American Electric Power (A.E.P.)

**Figure 6:**
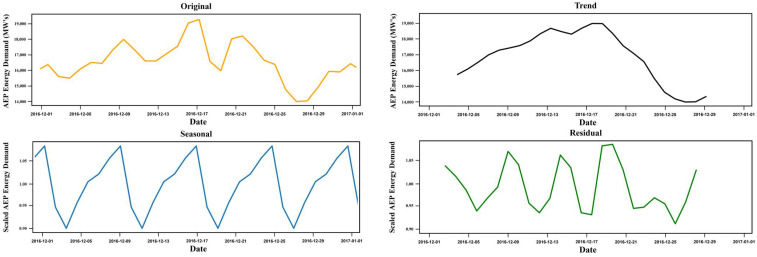
A.E.P. original and decomposed data for December 2016.

The dataset was obtained from the A.E.P., a power utility company covering 11 states in the United States and delivering energy to more than 5 million consumers. The collected data is in univariate time series and ranges from 31 December 2004 to 2 January 2018. Figure 6 represents the decomposition of the A.E.P. time series with hourly values representing the original, trends, seasonal, and residuals over the different periods. The figure highlights the pattern variations of electrical data measured in Megawatt’s (M.W.s) concerning other dates.

#### 3.1.2. New York City (N.Y.C.)

**Figure 7:**
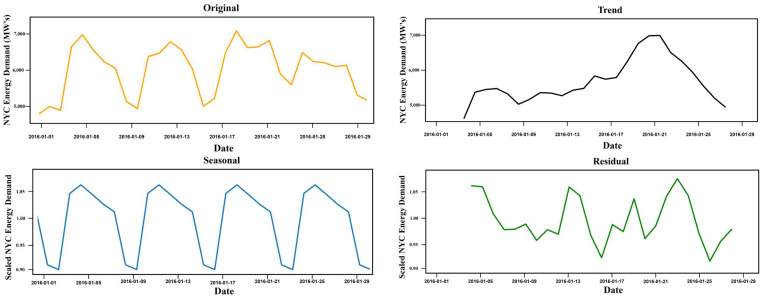
N.Y.C. original and decomposed data for January 2016.

The dataset was obtained from the N.Y.C., and it is a multivariate dataset that additional includes temperature and precipitation values and ranges from 2012 to 2017. Figure 7 represents the decomposition of the Dayton time series with daily values representing the original, trends, seasonal, and residuals over the different periods. The figure highlights the pattern variations of electrical data measured in Megawatt’s (M.W.s) concerning other dates.

#### 3.1.3. University Technology Petronas (U.T.P.)

**Figure 8:**
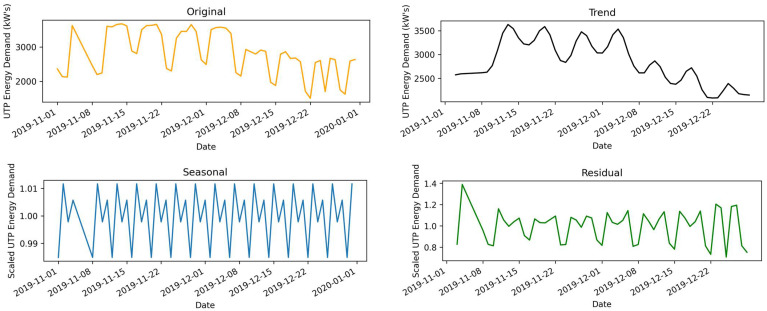
U.T.P. original and decomposed data for November and December 2019.

This data set was obtained from the U.T.P. gas district cooling (GDC) department, which provides the rest departments’ electrical services. This is a real data set comprised of hourly load demand for November and December 2019. The dataset is multi-variable with load demand, temperature, humidity, and wind speed variables. Figure 8 represents the decomposition of the A.E.P. time series with daily values representing the original, trends, seasonal, and residuals over the different periods. The presented figure highlights the pattern variations of electrical data measured in kilowatts (kW) concerning different dates.

### 3.2. Data Preprocessing

The datasets were preprocessed to eliminate the existence of any loss, noise, or insufficient data. Such values were eliminated considering respective appropriate algorithms. Later, the datasets were decomposed to attain the daily, weekly, and monthly hidden features. The features were then compared according to their correlations and influence on the output. The most optimized features were obtained, and the rest were discarded. However, during the data preprocessing, it was also noticed that weekly and monthly features had a similar impact on all three datasets. Moreover, the influence of specific parameters in combination was more significant than as individuals.

### 3.3. ARIMA

**Figure 9:**
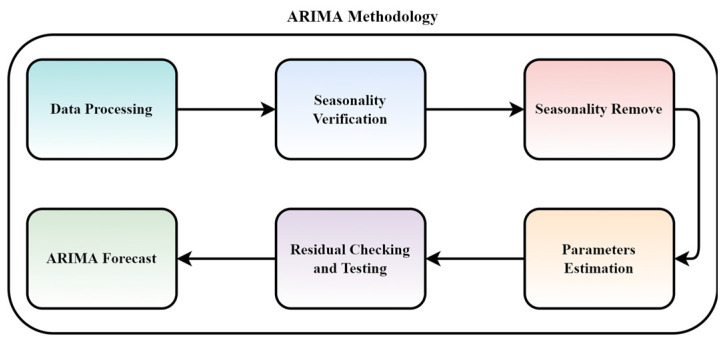
ARIMA methodology for the experimental framework.

Framework: The developed framework was to analyze the behavior of the ARIMA model on different datasets to calculate the performance change along with respective factors. Moreover, to observe the model deterioration when applied to different datasets, sectors, and applications. The model comprised modules performing data processing, verifying the seasonality, and removing the seasonality. Figure 9 presents the methodology followed in this framework.

Later, the model estimates the model parameters, checks for any residuals, and performs testing of the model. Finally, to produce a forecast considering all three datasets with parameter variations over the period.

### 3.4. ANN

**Figure 10:**
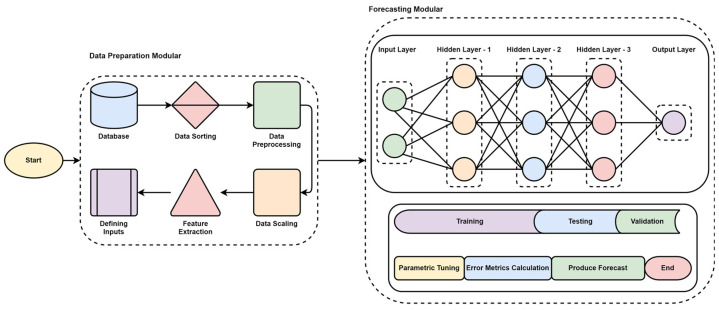
ANN methodology for the experimental framework.

Framework: The developed framework was based on the ANN architecture and is believed to provide the model performance and deterioration when input parameters vary with time. The process includes data preparation modular and forecasting modular. A brief pictorial is presented in Figure 10.

Data Preparation Modular: Databases provide the data, which are filtered for NaN and 0 values. Later the data is further preprocessed and scaled following the model requirements. Once the dataset is mounted, the input parameters are defined along with the data distribution with training, testing, and validation split with 70, 10, and 10, respectively. The input parameters are kept constant for initial simulations, and later the parameter changes are applied to observe the model behavior over time for the changed parameter. At this stage, the initial parameter change is limited to change in one parameter, including change in the *t* − 1 and *t* − 2 times the historical load. The scaled values are then processed for prediction.

Forecasting Modular: The forecasting modular comprises ANN architecture called neural networks and is inspired by the human brain. The neural networks are intelligent enough to learn from data to recognize the patterns, data classifications, and prediction of future values based on historical information. The architecture of ANN is discussed in detail in the literature review section.

The ANN forecasting modular comprises two input, three hidden, and one output layer. The parameter optimization is performed along with minimization of the loss function to avoid overfitting. An overfit function fails to uncover the hidden features, due to which it performs poorly. Different combinations for layers were utilized for optimal performance, but better results were generated using 27, 18, and 18 combinations for hidden layers and a dropout of 0.2. The model ran with 25 epochs at a batch size of 100 with a linear activation function known as ‘Relu.’

### 3.5. LSTM

**Figure 11:**
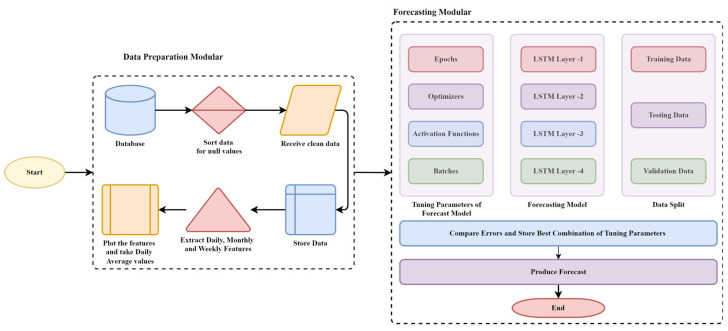
LSTM methodology for the experimental framework.

Framework: We developed the framework to examine the performance of existing E.L.F. models on different datasets, which explored the impact and factors affecting the accuracy of E.L.F. models and causing model deterioration. The process flow includes data preparation involving data cleaning, transformation, reduction, and feature extraction. Later it is introduced to the forecasting modular to produce the forecasting involving parametric tuning, error comparison, and forecast production, as depicted in Figure 11.

In this case study, we considered the datasets mentioned above. We also observed the impact and performance of the forecasting model before and after parametric tuning. We noticed the improvements in the forecasting results using the long short-term memory (LSTM) model. The methodology flow is distributed into two modules, namely, data preparation and forecasting. Figure 11 also presents the flow of methods.

The model performance is observed on the below basis:

1. Training Accuracy: We get the accuracy if we apply the model to training data.
2. Training Loss: It indicates how well the model fits the training data.
3. Validation Loss: It indicates how well the model fits new data.

Dataset Preparation Modular: The data are received from databases and forwarded for sorting to separate the null values from the dataset to improve its credibility. Later, the clean dataset is processed for data extraction of existing features and values such as load behavior daily, weekly, monthly, and yearly. Based on respective segments, the graphs are plotted to understand the load behavior concerning the different time ranges. For final processing, the received data is later considered for daily values, taken through average means. These values are scaled using Minmax Scaler. The absolute values are subsequently processed to forecast modular for further actions.

Forecasting Modular: The forecasting modular comprises sub-modules, namely, data split, model development, parametric tuning, error measurement and comparison, and production of load forecast.

Data Split: The dataset is split into training and testing, considering 70 and 30 percent ratios.

Model Construction: The construction of the LSTM model is comprised of 4 layers with unit values of 64, 32, and 16 with 25 epochs, the batch value of 32, Adams as an optimizer, and Relu as the current activation function. The drop out layer is set with a value of 0.3 and dense with a unit value of 1.

Parametric Tuning: The tuning of parameters is performed by selecting different combinations of values from Table 2, giving different sets of output with different per-centage of accuracy and error. Numerous simulations with different combinations were run to check the best suitable parameters throughout tuning. Every parameter impacted forecasting results that were run on the combinational basis of heads of parameters named in Table 2.

Error and Comparison: After every simulation is performed, the values of tuning parameters are changed, and each simulation has its product of error measurement, which is then stored to compare with other simulations to decide the optimal parameters for E.L.F.

Forecast: Once the different combinations of unique optimal parameters are obtained after continuous and regressive simulations. The results are compared, and the most effective combination of parameters is selected for producing the forecast.

### 3.6. Tentative Proposed Framework

Due to the rapid change in electrical dynamics and exponential growth in load demand, there is a need for continuous developments in machine learning and deep learning models. These models are capable of self-learning and adaptation to changes. Though the researchers are covering some dynamics in other fields for the transformation of models in classification applications [49,50,51], some propositions in regression works also exist [25,40]. Still, in regression, many features need to be incorporated when the task is specifically of electrical load forecasting to enable the model to be eligible for multi-modality or Smart Grid.

Since electrical data is acyclic and the demand continuously varies depending on different factors, including meteorology, region, power modality, events, etc. [6]. Therefore, a model which can integrate these changes when and where required in an online platform is required. Based on the conducted literature review, it is deduced that a model is needed which can incorporate the model changes based on meteorology, region, modality, application, and demand sector.

**Figure 12:**
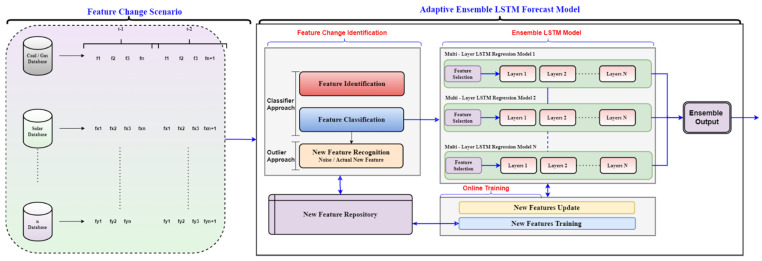
Adaptive Ensemble LSTM forecasting model framework with feature and modality change scenario.

The intuition behind this framework is to adapt the modality and variations in parameter and region features. In the proposed framework, the projected ensemble mechanism’s diversity helps to handle the new features’ possible arrival, specifically modality, parameters, and meteorological features. More precisely, this ensemble proposes a novel approach that contributes diversity to a simple yet effective ensemble system. Considering the existing research gaps in the proposed framework, we have integrated two different modules to introduce adaptive load forecasting. These modules incorporate the requirements to enhance and develop a novel adaptive model to fulfill the demand of modern load forecasting comprised of multi-modality generation and Smart Grid. Modular-1 consists of a data stream pipeline composed of different modalities and features, including the changing scenarios. Modular-2 is termed an adaptive ensemble framework modular, which is further sub-sectioned into three sub-modules: feature change identification, online training, variable weightage, and forecasting module. The detail of Figure 12 is presented below:

Data Stream Pipeline: The data stream pipeline is responsible for providing the data from input sources (different generation modalities) to the adaptive framework after observing the feature change. This module further consists of feature change scenarios which could be one or many concerning the case.

Feature Change Scenario: The first modular, namely the feature change scenario, takes input from the different generation modalities and sorts the features accordingly. This modular might experience a change of input features at the different time stamps from any or each input database involved. Thus, the set of features introduced at time *t* − 1 may experience a change at time *t* − 2 (shown in modular-1, feature change scenario). Such changes could occur as a completely new feature or a transformation of existing into new segments. Moreover, these changes could be experienced as single or multiple value changes.

Consequently, they cause the model deterioration in terms of its performance. Thus, examining the features and relevant changes at the initial stage is of utmost importance for the better performance of the model. Therefore, we capture these changes at an earlier stage to counter the impact on the model and work towards the timely provision of an appropriate solution.

Adaptive Framework: This modular comprises three further modules (feature change identification, online training, variable weightage, and forecast ensemble), which complete the requirement of an adaptive framework for a dynamic and challenging environment. The modules could be further described as below:

Feature Change Identification: The features received from the “feature change scenario” modular are now processed in the feature change identification modular. The modular is responsible for identifying the features and classifying them according to their presence or absence from the standard feature repository. The features resulting in negative compared to the standard feature repository are considered feature changes or new features. The modular involves feature classification and new feature recognition based on classified and outlier approach, later compared to the standard feature repository. Each feature is then identified, analyzed, and compared with the common feature repository to decide whether the feature is new or old. The features with favorable comparison travel to the old library whereas the features with negation are identified as new features and stored in the new feature repository.

This phenomenon of new feature identification repeats itself after the standard period, and these identified variables are then trained accordingly. The introduced features are updated in the library to feed the ensemble LSTM model for feature selection. These new features, unidentified in traditional load forecasting models, are responsible for the model’s performance deterioration. Therefore, we have integrated this modular to identify the feature change phenomenon in our framework. This will help the model analyze the input parameters with respect to changing modality and produce acceptable results compared to traditional models. 

Variable Weightage Module: Once the change in feature is identified and compared with the features library. The element is later weighted along with the existing and new library to compare the consideration weightage, and final variables are processed for dimensional reduction to filter only relevant and essential features to be considered for load forecasting. This function could be a single or a combination of multiple strategies to decide the variable weightage depending on the factors related to the application of forecasting.

Ensemble LSTM Forecasting Module: The forecasting module is built on a parallel concept working with multiple layers. It comprises parallel working LSTM modules that work in parallel to perform better forecasting to attain the desired results. LSTMs are believed to work better on large datasets, and due to their excellent performance, they produce acceptable results. However, we plan to introduce the modified LSTM network that will further enhance the capabilities and fit the requirement of multi-modality generation and S.G.s.

The existing models do not accept multiple input sources of generation or changes in input parameters at different time intervals (also stated in Figure 1 and described in traditional models). Moreover, they are not adaptive in terms of variation in feature, regions, application, classification, and identification of newly introduced parameters. Furthermore, no phenomenon of constantly updating the model concerning features and continuous feedback from output to update the model from the input is presented in the literature. The models reported in the literature require manual modification for every change required. Thus such models stand obsolete in dynamic environments. Therefore, we have proposed an adaptive framework that encompasses the adaptive characteristics that decide the feasibility of parameter selection and rejection according to the modality, which is based on the feature identification, classification, and recognition module. Such change identification records the introduction of new parameters and handles the repetition with the help of a feature repository that keeps a record. The data from the repository continuously updates the framework to update features and constantly update the training data, making it more rigorous and robust to keep track of changes and improve the performance accordingly. Thus, such characteristics of the proposed framework stand in a unique position compared to existing models.

Moreover, the considered data for implementation of the framework belongs to U.T.P. The data is susceptible and could not be released openly. However, we will discuss the attributes of the data and some system constraints. Due to high energy demand, efficient energy systems are required. A suitable solution for that is the gas district cooling (GDC) system using natural gas as a primary fuel source. The electricity generated at the U.T.P. comprises two units of gas turbines that support U.T.P. demand, internal usage, and chillers usage for the air-conditioning system. The data provided insights into peak hours ranging from 8:00 a.m. to 9:00 p.m., whereas off-peak hours are from 10:00 to 7:00 a.m. The change combination of parameters used before and after comprised date, time, historical load, temperature, wind speed, and humidity. 

Furthermore, the system under observation could experience constraints such as noise, insufficient data, false data, or data anomaly. The current framework consists of a module for dealing with noise, but more implications are required for further improvement. However, during the deployment of the framework, respective modules need to be actively deployed along with the framework.

Thus, this framework provides better results in a dynamic environment than existing load forecasting methods that stand obsolete in such environments, employing continuous updates on features, rigorous training of variables, and an ensemble of multiple multi-layer LSTM regression modules. Our future work is implementing the proposed framework to enhance load forecasting practically and lay the forecasting foundation for multi-modality generations.

## 4. Results and Discussion

### 4.1. Case Study-1 (Using ARIMA)

**Figure 13:**
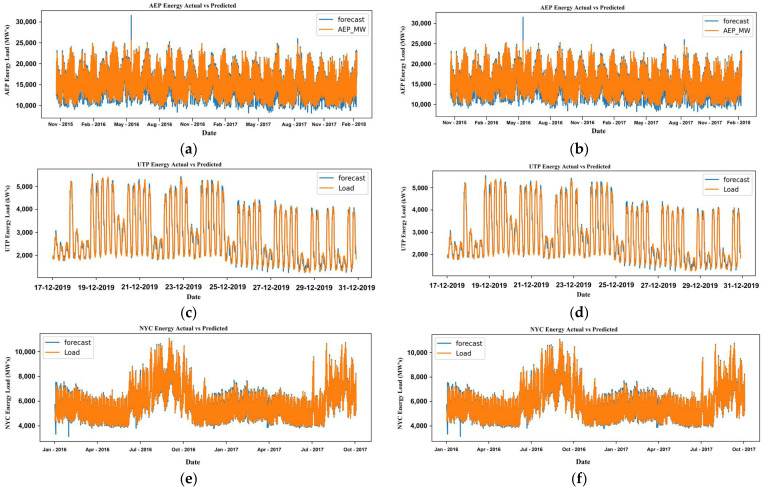
Actual and predicted results of A.E.P. (**a**,**b**), U.T.P. (**c**,**d**), and N.Y.C. (**e**,**f**) before and after parameter variation in provided dataset and forecasting model ARIMA.

We explored three different datasets with ARIMA and observed their behavior over time. We have presented the results of all these three different data sets before and after the variable change in Figure 13. Since the various parameters were introduced to the ARIMA model, unfortunately, the ARIMA model could not detect the features and kept its result based on a time series basis. Therefore, the before and after results were not much deviated. The metrics to observe and discuss the model’s performance are presented in Table 3. The ARIMA model could not detect the importance of newly introduced parameters, and the results remained more or less the same with minimal deviations. In Figure 13, (a) and (b) are represented for A.E.P., (c) and (d) for U.T.P., and (e) and (f) for N.Y.C. datasets referring to before and after figures, respectively.

### 4.2. Case Study-2 (Using ANN)

After reviewing several proposed models for load forecasting utilizing ANN [31,33,42,44], it is concluded that ANN methods have better forecasting accuracy than statistical methods. However, in this study, we utilized ANN and other forecasting models to understand the performance and behavior of models towards feature change scenarios. Most statistical methods stand obsolete in such a dynamic environment. Therefore, we utilized ANN and observed the performance of ANN in our feature change scenario. Despite having good accuracy results in several studies [42], the change remained unnoticed by ANN. We had to incorporate the shift physically into the model to improve its accuracy and determine its importance. 

**Figure 14:**
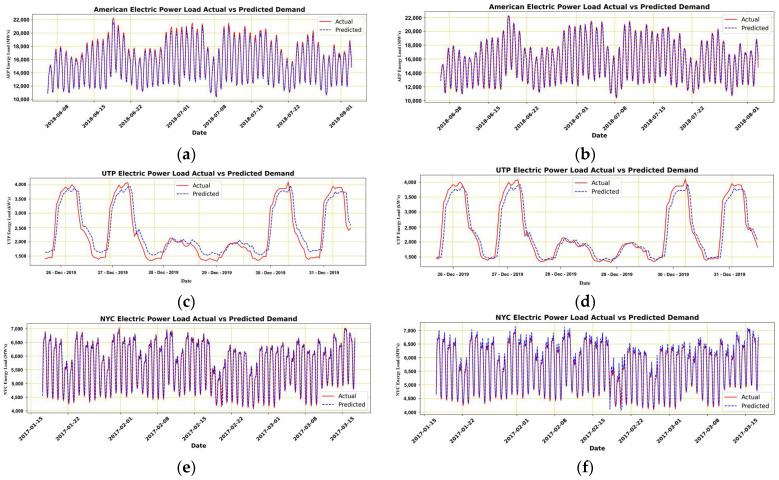
Actual and predicted results of A.E.P. (**a**,**b**), U.T.P. (**c**,**d**), and N.Y.C. (**e**,**f**) before and after parameter variation in provided dataset and forecasting model ANN.

Therefore, the results presented in Figure 14 show that despite good accuracy and precision, the ANN models do not respond to feature change scenarios automatically. In some situations, they even deteriorate their performance and provide poor forecasts. Due to this reason, we moved a step ahead and considered another forecasting model to observe its behavior. However, since the models’ do not tend to change their input behavior while monitoring the data, they tend not to change their set directive until defined. The inclusion and exclusion of parameters in the observed dataset have impacted the forecasting accuracy, but these changes were made to be observed manually. Some of the models that resulted in performance degradation are presented in Table 4. Figure 14, (a) and (b) are represented for A.E.P., (c) and (d) for U.T.P., (e) and (f) for N.Y.C. datasets referring to before and after figures, respectively.

### 4.3. Case Study-3 (Using LSTM)

**Figure 15:**
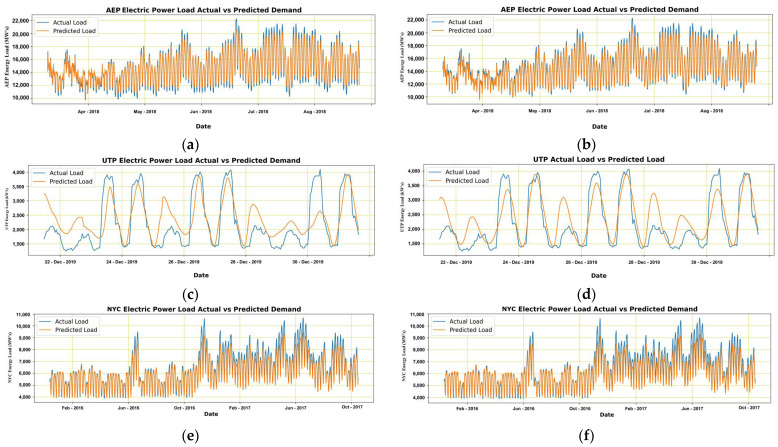
Actual and predicted results of A.E.P. (**a**,**b**), U.T.P. (**c**,**d**), and N.Y.C. (**e**,**f**) before and after parameter variation in provided dataset and forecasting model LSTM.

In this article, we reviewed studies that proposed LSTM models for electrical load predictions [12,21,29,30,31,32] and observed their performance. We have utilized the configuration of multivariate LSTM to cater to the maximum number of variables. However, there still exists room for improvement. Figure 15 provide LSTM models’ performance before and after parametric change for A.E.P., U.T.P., and N.Y.C. datasets, respectively. 

The dataset of A.E.P. and N.Y.C. were distributed over two years and are open-source datasets, whereas the U.T.P. dataset is a real environment dataset available for only two months. The before variables included DateTime and load, and the after variables included temperature along with earlier variables. The LSTM model was first run with different combinations of activation functions and optimizers and tested on distinct varieties described in the modular forecasting section to optimize and tune the parameters for optimum results.

It is to be noted that when we introduced a new change in the dataset, it was unrecognizable in 2 cases. In contrast, the third case decreased its output accuracy after introducing a new parameter. Therefore, to measure the importance of this new variable, we introduced it to the model manually and measured the error percentage before and after the introduction of the new variable. The introduction of a new variable has improved the forecasting accuracy in all three cases. Therefore, it is deduced that the models cannot notice the presence and importance of any newly introduced variable until or unless it is present in the model or altered manually, which affects the model performance. Figure 15, (a) and (b) are represented for A.E.P., (c) and (d) for UTP, and I and (f) for N.Y.C. datasets referring to before and after figures, respectively.

The experimental results are presented in Table 3, Table 4 and Table 5 numerically for ARIMA, ANN, and LSTM. The table provides the model’s performance before and after the context of variable change, including MAPE and R2 values as performance metrics. We can notice that the minimum value of MAPE can be observed in simulations performed under the “After” label, which means introducing new parameters manually in the model. However, the maximum MAPE values can be kept under the “Before” label, which indicates the traditional parameters results. Moreover, it is to be noticed that the introduction of new parameters was also made in the dataset under the “Before” label. Still, they remained unnoticed, and some models even depreciated more than the presented results. Furthermore, a similar situation is experienced by the R^2^ score; however, the highest score is considered better, which shows the data fit percentage.

When we compare the performance of different models over the A.E.P. dataset, it is evident from the results that the ANN model has outperformed ARIMA, and LSTM results were the nearest. A similar situation was encountered in the N.Y.C. and U.T.P. datasets as well. The performance of models can be graphically observed in Figure 13, Figure 14 and Figure 15. The A.E.P. and N.Y.C. data had multiple years of hourly load due to which they were observed to be well-trained; however, the data of U.T.P. was for just two months, due to which the models were unable to perform sufficiently on that. It is to be noted that the nature of U.T.P. data is different in comparison to others; therefore, modeling that data requires more precision.

However, it is evident from the presented results that the performance of E.L.F. models deteriorates over time with different changes and is also dependent on the methodology adopted at development time. Moreover, it is also evident from presented facts and literature that the S.G. and D.G.M. environments are dynamic, and such traditional models will stand obsolete. Furthermore, the literature’s proposed adaptive models have limitations discussed in Table 1. 

Therefore, an adaptive model framework is proposed in this study as future work, which will adhere to the adaptability limitations of features, parameters, modality, region, industry, utility, power sector, D.E.R.s, I.P.P.s, and application environment. The presented framework is believed to be significantly crucial in S.G. and D.G.M. environments where the data is highly nonlinear with dynamic behavior and consists of multiple different generation resources having various factors which impact the prediction accuracy of E.L.F. model.

## 5. Conclusions

STLF has been of utmost importance for implementing S.G.s and integrating D.G.M.s. Several factors challenge accurate load forecasting, including meteorological and production sources. Moreover, the models that can incorporate the characteristic change over time and are adaptable are significant. Since the traditional models are obsolete in dynamic and real-time environments. Several models have been discussed, but industry, residential, utility, S.G.s, D.G.M.s, and D.E.R.s are the environments where an efficient adaptive E.L.F. model is of paramount significance.

This paper investigated the limitations of recent models. It proposed a framework by combining a classifier approach and outlier detection to improve the feature change and identification scenario significantly. An LSTM ensemble working with feedback and continuous feature update will adapt the model to the outer environment, parameter, modality, and scenario change. Minimal studies explore or employ the online E.L.F. learning techniques and require sufficient modifications and diversification for dynamic environments.

Despite the described advantages of the presented framework, some limitations include defining significant differences in training and forecasting in real-time due to non-stationarity and continuous data input. Furthermore, some challenges that we would like to address in future works include analysis of injection of unlabeled and labeled data, defining boundaries for noise removal modular, different parametric tuning incorporation, and defining standards for inclusion or exclusion of parameters.

By analyzing the experiments performed above, the following conclusions can be drawn:

- Change in input variables deteriorates the performance of load forecasting models.
- Input variables impact the rate of error, which is inconsiderable for the S.G. and D.G.M. environment.
- Change in input variables impacts error percentage depending on the parameter introduced.
- Consideration of month and week as variables has no significant impact on considered datasets.
- Meteorological variables profoundly impact the E.L.F. model performance, and no standard is defined for such parameters since the parameter change concerning region, sector, modality, and application.
- Traditional models stand obsolete in S.G. and D.G.M. environments. Thus, real-time adaptive models are required.

## Figures and Tables

**Table 1:** Summary of the comparative study of a+daptive E.L.F. models based on their strengths.

Ref.	Models	Adaptive Strengths					Remarks	
		A	F/P	M	Rt	Re/S		
[15]	ATDGM	**	*	X	*	X	Inefficient to handle the existence of complex-nonlinearity. Exhibits periodicity due to incurred errors. Morbidity of the matrix occurs due to the large magnitude of observations.	
[31]	ABPA	**	*	X	*	X	Segregation of train, validation, and forecast is required. Furthermore, B.P.A.s are slow and have unreliability regarding new environments. They cannot quickly adapt their parsing and need an alternative.	
[32]	ANFIS-LSSVM	**	*	X	*	X	It is not adaptable to real-time and feasible for only pattern-based scenarios due to its dependency on K.N.N. Noise sensitivity exits, leading to unacceptable results.	
[33]	FM-MLP	**	X	X	*	X	Produce errors with changes in patterns on special days and events. Unresponsive to change behavior in real-time is highly likely in the S.G. and D.G.M. environment.	
[34]	I.L.R.	**	X	X	*	X	The proposition is based on a linear approach that highly unfits to satisfy the requirement of S.G. and D.G.M. to their continuous nonlinearity and existence of noise and insufficient data. Thus, the model does not suit other generation sources.	
[35]	SGD-RNN	**	*	X	**	X	The presented model has claimed to perform adaptive forecasting with outliers’ resistance; however, the strategy dealing with outlier and change points has severe complexity leading the model to deviate from actual predictions. Moreover, the model lacks the integration of new parameters and modality, which is an integral part of S.G. and D.G.M.	
[40]	ARIMA-RNN	**	*	X	**	X	Rolling ARIMA sliding window is considered to develop a real-time environment that is error and noiseless, thus creating an ideal environment not comparative with a real-time environment. Moreover, ARIMA in a highly nonlinear environment will produce more errors resulting in more untrue sequences, producing more unrealistic data. Thus, on the contrary, model performance could be considered acceptable, but it will deviate a lot in a real environment, leaving the model to be unacceptable for S.G. and D.G.M.	
[41]	BC-MMT	**	X	X	*	X	The proposal focused on HPC systems dealing with data disruptions during hardware and software up-gradation or degradation only and adapts accordingly. However, in comparison, the scenarios verily differ. Therefore, unacceptable in S.G. and D.G.M.	
[42]	P.A.R.	***	X	X	***	X	The model has improved forecast with great adaptability but has limitations regarding parameters, modality, and region expanding to industry, utility, and application. However, the model has produced many forecasts. The modifications could result in considering the model for S.G. and D.G.M.	
[43]	R.N.N.	***	X	X	***	X	The proposed adaptive R.N.N. produced better predictions than other models; however, the online models deal with real-time environment data with no significant parameter or modality changes. However, the model has produced acceptable results, but integrating with S.G. and D.G.M. requires significant modifications as the relationship modeling between model behavior to parameters and occurrence of concept drift.	
[44]	AMLP	**	X	X	X	X	The presented results have a high error rate and adaptability limitations regarding parameters, modality, and region expanding to industry, utility, and application.	
[46]	MARS	**	*	X	X	X	MARS has produced efficient results but dealing in a single generation data type flexes the model performance. However, the effects on real-time deviate as the environment changes.	
[47]	CLFIF-IL-ChOA	**	**	X	**	X	The model is built constructively to deal with parameterization and has produced effective forecasts. However, parameter adaption is not only a function in S.G. and D.G.M. Therefore, applicable modifications are also required.	
[48]	R.L.	**	X	X	**	X	The proposed model performed prediction interval (P.I.) for finding uncertainty in distribution systems. The employed R.L. strategy is efficient; however, the behavior of S.G. and D.G.M. environments are comparatively different from P.I.s.	
[49,50,51]	CNN	***	***	X	***	X	The study proved to be a benchmark for several studies in classification. However, the S.G. and D.G.M. environment are based on regression. Therefore, modifications are required to improvise the model and devise it according to regression.	
-	Proposed’ framework	***	***	***	***	***	The proposed framework encompasses the characteristics that feasibly adjust the parameter selection and rejection based on modality. It is based on the feature identification, classification, and recognition module that records the new parameters. The model is continuously updated, making it more rigorous and robust to keep track of changes and improve the performance accordingly.	
Strengths Abbreviations								
*	Good		A	Adaptability			R_t_	Real-time
**	Better		F	Feature			R_e_	Region
***	Best		P	Parameter			S	Seasonality
X	Feature does not exist		M	Modality				
Abbreviations of models used in Table 1.								
ABPA	ANN-back propagation Algorithm		AMLP	Adaptive multi-layer perceptron			ANFIS-LSSVM	Adaptive neuro-fuzzy inference system least-squares support vector machine
AT DGM	Adaptive time-varying discrete grey model		CNN	Convolution NN			BC-MMT	Bayesian-Changepoint Moment-Matching Transformation
FM	Forecasting monitor		HPC	High-performance computing			CLFIF-IL-ChOA	Composite Linear Fractal Interpolation Function with Iterative Learning and Chimp Optimization Algorithm
I.L.R.	Integrated linear regression		R.L.	Reinforcement learning			MARS	Multivariate Adaptive Regression Splines
P.A.R.	Passive aggressive regression		R.N.N.	Recurrent neural networks			SGD-RNN	Stochastic Gradient Descent—RNN

* = Good, ** = Better, *** = Best.

**Table 2:** Parametric tuning components and their respective variational combination table.

Epochs	Optimizers	Activation Functions	Batches
5/10/25	Adams	Sigmoid/Tanh/Relu	8/32/64
5/10/25	RMS Prop	Sigmoid/Tanh/Relu	8/32/64
5/10/25	Adadelta	Sigmoid/Tanh/Relu	8/32/64
5/10/25	Adagrad	Sigmoid/Tanh/Relu	8/32/64
5/10/25	Stochastic Gradient Descent	Sigmoid/Tanh/Relu	8/32/64

**Table 3:** ARIMA model performance on A.E.P., N.Y.C., and U.T.P. datasets before and after parametric variations.

Model	Dataset	MAPE		R^2^ Score	
		Before	After	Before	After
ARIMA	AEP	21.5	19.3	0.54	0.67
	NYC	28.5	22.1	0.48	0.50
	UTP	38.2	36.4	0.44	0.47

**Table 4:** ANN model performance on A.E.P., N.Y.C., and U.T.P. datasets before and after parametric variations.

Model	Dataset	MAPE		R^2^ Score	
		Before	After	Before	After
ANN	AEP	3.6	1.79	0.94	0.99
	NYC	3.2	1.89	0.96	0.99
	UTP	24.6	8.1	0.55	0.89

**Table 5:** LSTM model performance on A.E.P., N.Y.C., and U.T.P. datasets before and after parametric variations.

Model	Dataset	MAPE		R^2^ Score	
		Before	After	Before	After
LSTM	AEP	3.7	2.7	0.92	0.95
	NYC	5.7	5.4	0.87	0.89
	U.T.P.	24	20.654	54	0.65

## Data Availability

Restrictions apply to the availability of U.T.P. data. The data were obtained from University Technology Petronas (U.T.P.) gas district cooling (GDC) department, which provides the electrical services to the rest of the departments. Other datasets are available online on the Kaggle website at https://www.kaggle.com/dataset (accessed on 3 January 2022).
